# Nicotinamide Riboside Neutralizes Hypothalamic Inflammation and Increases Weight Loss Without Altering Muscle Mass in Obese Rats Under Calorie Restriction: A Preliminary Investigation

**DOI:** 10.3389/fnut.2021.648893

**Published:** 2021-09-13

**Authors:** Josimar Macedo de Castro, Dirson João Stein, Helouise Richardt Medeiros, Carla de Oliveira, Iraci L. S. Torres

**Affiliations:** ^1^Postgraduate Program in Medicine: Medical Sciences, School of Medicine, Universidade Federal do Rio Grande do Sul, Porto Alegre, Brazil; ^2^Laboratory of Pain Pharmacology and Neuromodulation: Preclinical Studies, Experimental Research Center, Hospital de Clínicas de Porto Alegre, Porto Alegre, Brazil; ^3^Animal Experimentation Unit, Grupo de Pesquisa e Pós-Graduação, Hospital de Clínicas de Porto Alegre, Porto Alegre, Brazil; ^4^Faculdade São Francisco de Assis (UNIFIN), Porto Alegre, Brazil

**Keywords:** nicotinamide riboside, caloric restriction, obesity, neuroinflammation, muscle mass, skeletal muscle tissue, hypothalamus, brain

## Abstract

Obesity treatments, such as calorie restriction (CR), eventually lead to muscle wasting and higher rates of neuroinflammation, whereas hypothalamic inflammatory conditions impair body weight (BW) control. Nicotinamide riboside (NR) has been proposed against obesity but with little evidence on skeletal muscle tissue (SMT) and neuroinflammation. Therefore, we aimed to investigate the effects of CR on SMT and on hypothalamic inflammatory biomarkers in obese adult male Wistar rats, and whether NR supplementation alone or in combination with CR affects these parameters. Obesity was induced in rats through a cafeteria diet for 6 weeks. After that, a group of obese rats was exposed to CR, associated or not associated with NR supplementation (400 mg/kg), for another 4 weeks. As a result, obese rats, with or without CR, presented lower relative weight of SMT when compared with eutrophic rats. Rats under CR presented lower absolute SMT weight compared with obese and eutrophic rats, in addition to presenting elevated hypothalamic levels of TNF-α. NR supplementation, in all groups, enhanced weight loss and increased relative weight of the SMT. Furthermore, in animals under CR, NR reversed increases TNF-α levels in the hypothalamus. In this study, these data, although succinct, are the first to evidence the effects of NR on SMT and neuroinflammation when associated with CR, especially in obesity conditions. Therefore, this provides preliminary support for future studies in this investigative field. Furthermore, NR emerges as a potential adjuvant for preventing muscle mass loss in the weight loss processes.

## Introduction

Presenting global epidemic proportions, obesity is a chronic metabolic disease associated with serious health problems (1). Ideally, its treatment is aimed at a gradual loss of body weight (BW), mainly through the reduction in body fat. In this sense, both physical activity and calorie restriction (CR) are the standardly used clinical practices (1). However, CR regimens particularly can lead not only to reduced body fat but also muscle protein breakdown, which subsequently causes muscle wasting (2, 3). Loss of muscle mass is a considerable risk factor for bone fractures, cardiovascular problems, and metabolic dysfunctions. In addition, complications in skeletal muscle tissue (SMT) impair the whole-body bioenergetic metabolism and hinder the process of weight loss (4). Therefore, a healthy weight loss strategy should have STM as a focus as well.

Changes in body composition, especially in SMT, are directly coordinated through the brain (5). In the central nervous system (CNS), hypothalamic neurons are mainly responsible for the management of eating behavior and energy expenditure. Additionally, hypothalamic stimuli inherent to whole-body energy homeostasis (e.g., fasting or feeding) or local neurochemical insults (e.g., inflammation) can unbalance neuronal circuits and affect both BW loss and lean mass maintenance processes (5–7). Moreover, hypothalamic inflammatory factors are known to be closely associated with morbid obesity and muscle syndromes associated with muscle wasting and weakness (7).

Nutritional compounds combined with conventional weight loss interventions have been widely encouraged against obesity, especially if they simultaneously bring benefits to SMT (8). In line with this, nicotinamide riboside (NR), a dietary compound analogous to vitamin B3, has been shown in recent experiments as effective for weight loss and SMT health maintenance (9–11). We have previously shown that supplementation with NR positively modulates obesity-related parameters in both obese rats and obese rats under CR, including reduced BW gain and reduced adiposity (12), unaware of whether this process affected SMT or neuroinflammatory parameters. As studies on this topic are really scarce, with no clinical or preclinical evidence reporting its potential effects on muscle mass or neuroinflammatory events when associated with a CR strategy under obesity conditions, in this study, we aimed to investigate whether NR supplementation, combined or not with CR, affects muscle mass and hypothalamic inflammatory parameters of rats with obesity induced by a hypercaloric diet model.

## Materials and Methods

### Animals and Ethical Procedures

The data presented in this study were obtained and continued from our previous experiments (12). A full version of the methods is also published in this study. In summary, 52 adult male Wistar rats (60 days old; weight ±250 g) were used, and three to four animals were housed per polycarbonate cage, with sawdust-covered floors and enriched using cardboard tubes. Animals were maintained under strictly controlled environmental conditions (12-h light–dark cycle, temperature 22 ± 2°C, air humidity 50 ± 5%, and water and rodent chow *ad libitum*). All experiments and procedures were approved by the Institutional Committee for Animal Care and Use of the Hospital de Clínicas de Porto Alegre (GPPG-HCPA protocol #2018-0049). The experimental protocol complied with the ethical and methodological standards of the ARRIVE guidelines (13).

### Experimental Design

**Figure 1:**
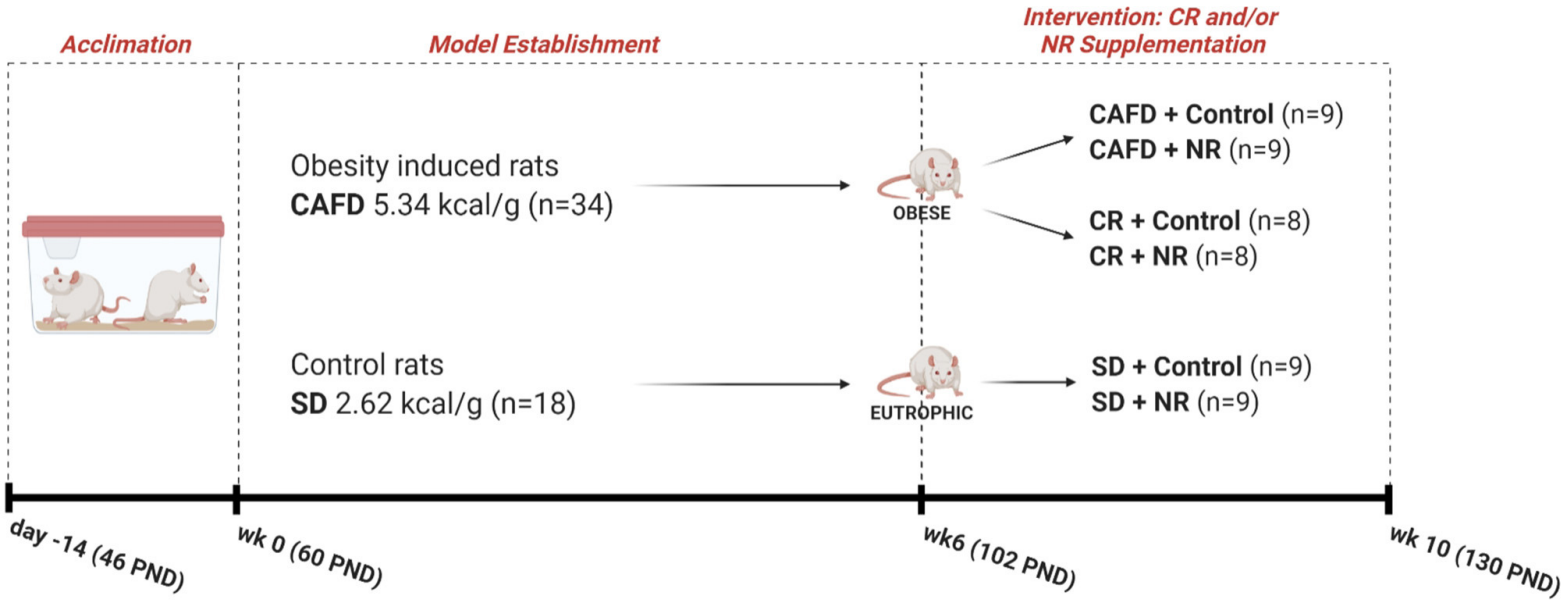
Experimental design. SD, standard diet; CR, caloric restriction; CAFD, cafeteria diet; NR, nicotinamide riboside; kcal, kilocalorie; g, gram; wk, week; PND, postnatal day; *n*, total of animals.

The study was carried out in two phases, as follows: (1) period of induction to obesity (for 6 weeks) where one group of animals was exposed to cafeteria diet (CAFD) and another to a standard diet (SD); (2) period of intervention with NR supplementation and/or CR (for 4 weeks). In the intervention period, the animals were subdivided into three groups (CAFD, CR, and SD). Additionally, these three subgroups were further subdivided into the vehicle and NR-supplemented animals, resulting in six subgroups (Figure 1). Noteworthy, CAFD for 6 weeks was efficient in inducing obesity (12). CAFD is a robust and translational obesity model that aims to offer animals the same and main obesity-generating hypercaloric foods in humans, for example, flavored snacks, stuffed crackers, processed meats, and sugar-sweetened beverages (14). The constituent foods of CAFD were freely distributed to the animals and were replaced daily with fresh and new foods and beverages. The dietary pattern was changed weekly, being replaced by foods with new flavors, odors, and shapes, in order to avoid adaptation and to maintain the novelty aspect of the diet. CR is defined as a reduction in dietary calories usually consumed without restricting micronutrients (e.g., vitamins and minerals) (2). Thus, the CR protocol consisted of replacing a hypercaloric diet (CAFD) with a standard diet (SD) for 4 weeks, starting immediately after the establishment of obesity. This process promoted, on average, a 62% reduction in calories ingested when compared with calories ingested during CAFD access (12). Nutritional characteristics of both diets are presented in Table 1, and complete nutritional information in Supplementary Material. Regarding NR (400 mg/kg) (15, 16) or control (vehicle/distilled water 0.5 ml) supplementation, both were administered orally, aiming at the translational aspect. This intervention was performed after the obesity induction period. Information about experimental groups, *n* per group, and the experimental timeline is presented in Figure 1.

**Table 1:** Comparative nutritional description of experimental diets.

	**Standard diet (SD)**	**Cafeteria diet (CAFD)**	**Δ Difference (%)**
Total energy (kcal = kJ)	26.2 = 109.6	53.4 = 223.4	103.8
Kilocalories per gram	2.62	5.34	103.8
Total Fat g (%)	0.40 (4.0)	2.44 (24.4)	510.0
Total carbohydrate g (%)	3.45 (34.5)	6.25 (62.5)	81.1
Dietary fiber g (%)	0.70 (7.0)	0.14 (1.4)	−80.0
Protein g (%)	2.19 (21.9)	1.25 (12.5)	−42.9
Sodium mg (%)	27.0 (0.27)	75.0 (0.75)	177.7
Total vitamins and minerals mg (%)	3.50 (0.03)	*	NA
Other food additives g (%)	3.22 (32.2)	^*^	NA

*Values equivalent to a 10 g portion consumed by animals, averaged across the study. Nutritional values calculated based on commercial product information*.

*Kcal, kilocalorie; kJ, kilojoule; g, gram; %, percentage; mg, milligram; Δ, delta denominator (indicates in percentage how much CAF values are greater or lower than SD values)*;

* unreported or established values; NA, not applicable. Formula: Δ $difference=\frac{\left(CAFD\;value-SD\;value\right)*100}{SD\;value}$.

### Biochemical and Weight Analysis

The animals were weighed weekly throughout the experiment. Delta weight gain was calculated using BW value from week 6 to 10 by the formula: Δ* weight gain* = ((*BW at week*10 − *BW at week*6)*100). Relative weight of gastrocnemius was calculated using final BW (week 10) and the absolute weight of the structure, by the formula: $relative\;weight=\left(\left(\frac{BW\;at\;week10}{absolute\;weight\;of\;gastrocnemius}\right)*100\right)$. Twenty-four hours after the end of the experiment, animals were euthanized by decapitation, and the biological samples were collected. SMT, more precisely, total gastrocnemius (from origin to insertion) of the left lower limb, and total hypothalamus were gently dissected, immediately weighed (semianalytical balance), and subsequently frozen at −80°C. The hypothalamus was homogenized using a handheld homogenizer with a 1:10 protease inhibitor cocktail (Sigma^®^ #P8340) and centrifuged for 5 min at 10,000 rpm using the supernatant for the technique. The levels of hypothalamic TNF-α (DY510) and IL-10 (DY522) were determined by sandwich ELISA using monoclonal antibodies (R&D Systems; Minneapolis, MN). Total protein was determined by Bradford's method (17) using bovine serum albumin as standard. Data are expressed in pg/μg of protein.

### Statistical Analysis

The variable normality was verified by Shapiro–Wilk tests and histograms. Absolute weight and relative weight of gastrocnemius and delta weight gain were analyzed by the two-way ANOVA parametric test (diet and supplementation factors). Hypothalamic TNF-α and IL-6 levels were analyzed by non-parametric tests; for diet purposes, Kruskal–Wallis test, and for NR supplementation purposes, Mann–Whitney test. Data were examined using SPSS Software (version 20) (IBM Corporation, Somers, NY, USA) and expressed as mean ± SEM, considering significant differences with *P* < 0.05. F-statistics (F), mean of difference (MD), confidence interval (CI), chi-square (χ^2^), and U-statistics (U) were reported for effect size purposes.

## Results

### Effects of CR and NR on Delta Weight Gain

**Figure 2:**
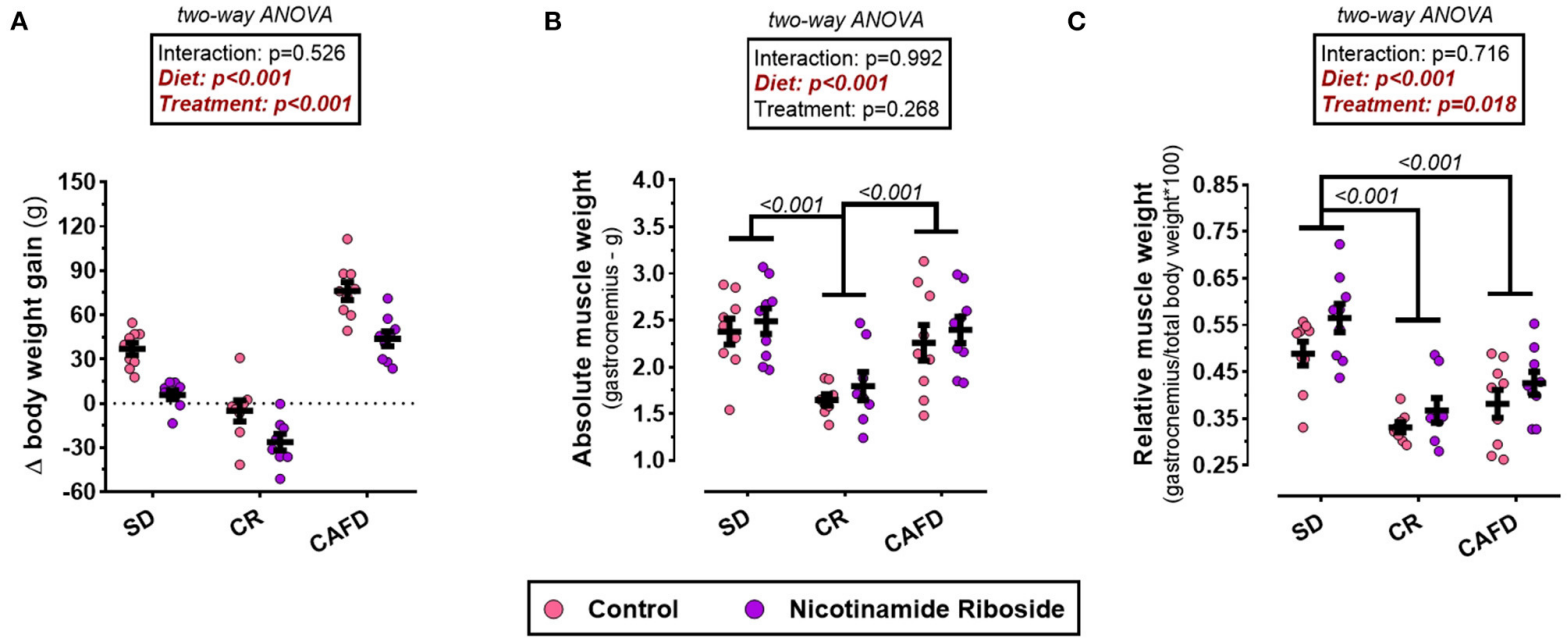
Effects of caloric restriction and/or NR supplementation on gastrocnemius muscle and body weight gain. **(A)** Δ weight gain. **(B)** Absolute weight of gastrocnemius. **(C)** Relative weight of gastrocnemius. SD, standard diet; CR, caloric restriction; CAFD, cafeteria diet; g, gram; Δ, delta denominator (indicates in grams how much the body weight of the animals increased or decreased at the end of 4 weeks of intervention).

According to the two-way ANOVA, there was no interaction between the factors diet and supplementation on variable Δ weight gain. The CAFD group when compared with the animals of the SD group showed an increase in BW gain [MD 38.5 g, CI95% (25.7–51.3), *P* < 0.001]. The intervention with CR promoted a significant loss of BW compared with the CAFD and SD groups, respectively [MD −75.5g, CI95% (−62.3 to −88.8), *P* < 0.001; MD −37.2 g, CI95% (−50.2 to −23.7), *P* < 0.001]. On the other hand, NR supplementation delayed and reduced Δ weight gain, respectively, in the SD and CAFD groups. Moreover, it maximized BW loss in the CR group [*F*_(1, 46)_ = 42.984; *P* < 0.001; Figure 2A].

### Effects of CR and NR on Absolute and Relative Weight of Gastrocnemius Muscle

According to the two-way ANOVA, there was no interaction between the diet and supplementation on the variables and relative and absolute weight of the gastrocnemius muscle. The absolute weight of the gastrocnemius was lower in the CR group, compared with both SD and CAFD groups [respectively, MD −0.60 g, CI95% (−0.97 to −0.24), *P* < 0.001; MD −0.71 g, CI95% (−1.07 to −0.34), *P* < 0.001]. The relative weight of the gastrocnemius was lower in the CR and CAFD groups compared with the SD group [respectively, MD −0.17 g, CI95% (−0.24 to −0.11), *P* < 0.001; MD −0.12 g, CI95% (−0.18 to −0.60), *P* < 0.001]. NR supplementation increased the relative weight of gastrocnemius in all groups [*F*_(1, 46)_ = 6.045; *P* = 0.018]. On the absolute weight, NR supplementation had no significant effect; Figures 2B,C.

### Effects of CR and NR on Cytokines in the Hypothalamus

**Figure 3:**
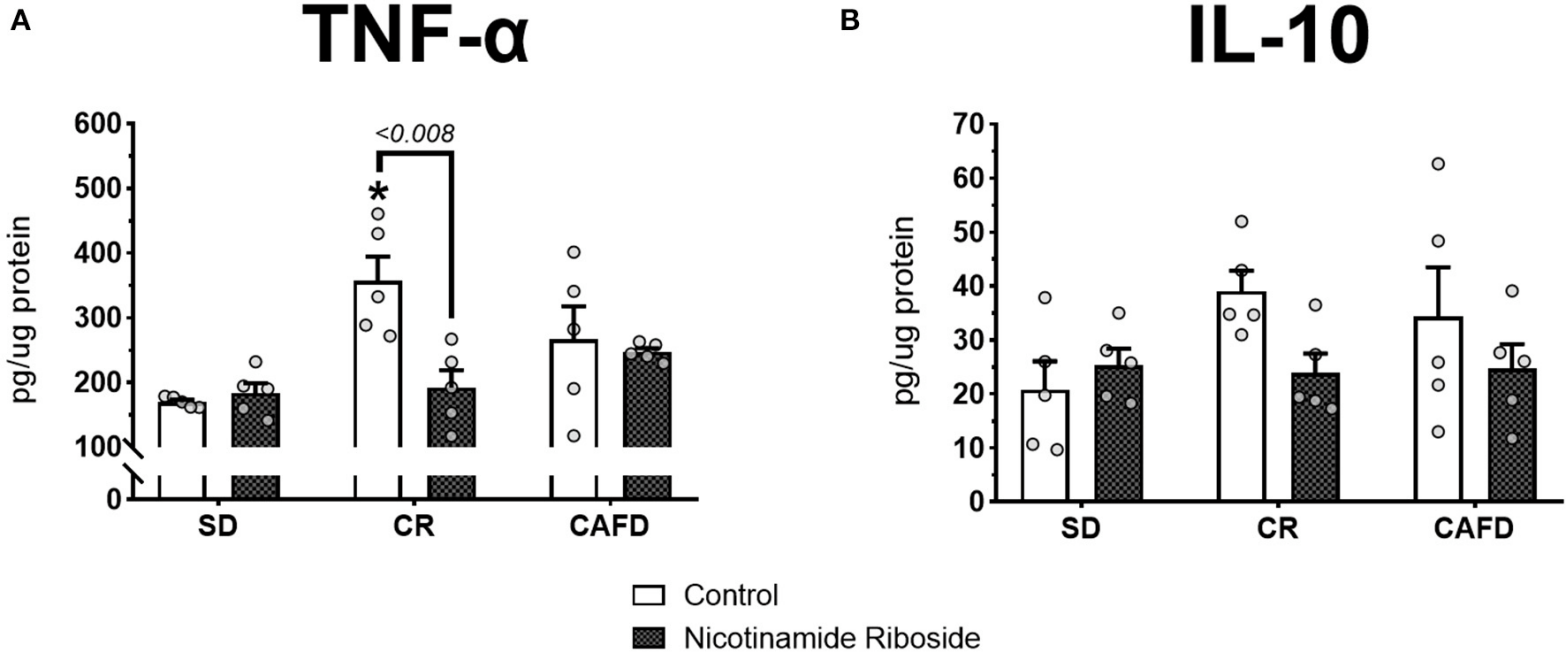
Effects of caloric restriction and/or NR supplementation on hypothalamic cytokine levels. **(A)** TNF-α. **(B)** IL-10. SD, standard diet; CR, caloric restriction; CAFD, cafeteria diet; g, gram; pg, picogram; μg, microgram. ^*^P < 0.05, CR+control compared to SD+control.

According to the Kruskal–Wallis test, the CR group presented higher hypothalamic TNF-α levels compared with the SD group [${\text{χ}}_{\left(2\right)}^{2}$ = 7.280, *P* < 0.026]. There were no significant TNF-α level differences between the other groups nor for hypothalamic IL-10 levels. According to the Mann–Whitney test, NR supplementation reversed the increase in hypothalamic TNF-α levels in the CR group (*U* = 25.000, *P* < 0.008]. Other significant effects with NR supplementation were not observed; Figures 3A,B.

## Discussion

As far as we know, these preliminary data are the first in the literature to evidence the combined effects of NR and CR on muscle tissue, especially in obesity conditions. We have previously shown that 28 days of CR in rats with obesity effectively reverses obesogenic indicators (12). In fact, CR is undeniably effective in the short term and medium term to reduce BW and adiposity (2). However, the deficit in energy intake often culminates in muscle wasting (2, 3). In addition, CR is known to inhibit the mechanistic target of rapamycin (mTOR), an energy-sensing cell signaling pathway that initiates cascades of anabolic reactions involved in muscle protein synthesis (18). These signals and reactions are essential for muscle mass both to stimulate hypertrophy and to prevent atrophy (4). Besides that, a low protein intake unintentionally reduces the availability of essential amino acids that are crucial substrates for mTOR signaling and muscle protein synthesis (19). In this line, before being exposed to CR, the animals of this group consumed 42% less protein than the control animals. Therefore, in an overview, the reduction in both absolute and relative weight of the gastrocnemius in animals under CR may be a reflex of muscle mass loss or delay in its gain, probably resulting from impairments in anabolic cellular mechanisms.

Another potential mechanism is related to the CNS. In the hypothalamus, CR caused a significant increase in TNF-α cytokine without altering IL-10 cytokine levels. TNF-α is usually involved in proinflammatory processes whereas IL-10 is responsible for inhibiting them (20, 21). In this sense, it is plausible to suppose that these animals have an unbalanced inflammatory profile in the hypothalamus induced by CR. Proinflammatory mediators can alter the brain neurochemistry, influencing peripheral systems (20). Particularly in the hypothalamus, these signs are closely linked to alterations in BW and in muscle dysfunctions (5, 6). Furthermore, hypothalamic inflammatory reactions drive the sympathetic nervous system and the hypothalamus–pituitary–adrenal axis to increase, respectively, systemic levels of epinephrine and glucocorticoids (4, 6, 22). These processes, uninterruptedly, stimulate muscle protein breakdown, inducing SMT atrophy (6). Thus, this reinforces our view that animals under CR have lost lean mass or have had a delay in their gain.

We observed that in all NR-supplemented groups, there was a significant increase in the proportion of gastrocnemius in relation to BW. Noteworthy, in the previous study we showed that NR prevented BW gain and adiposity in these three dietary conditions (12), which would partially justify this observation regarding relative muscle weight. Accordingly, if NR can maintain lean mass weight while substantially reducing fat mass, it supposedly presents a potential benefit for SMT. Even with scarce data in the literature, it has recently been reported that aged mice exposed to a diet containing NR exhibited an increase in the diameter of slow-twitch muscle fibers, despite not being stated if there was an effect in the total mass of the structure (23).

In general, NR is a potent nicotinamide adenine dinucleotide (NAD) enhancer, and its biological effects may be inherent to this mechanism (9, 24). NAD is a key coenzyme for muscle cells, both for energy metabolism and muscle contraction (25). Furthermore, it serves as a co-substrate for post-translational modifications (for example, mono/poly-ADP-ribosylation and deacetylation) that influence the health and maintenance of SMT (25–28). We recognize that the lack of histological data, particularly to determine the cross-sectional area of skeletal muscle fibers, and of molecular data, aiming to quantify NAD and its derivatives, are limitations of this study. Collectively, these analyzes would lead to more conclusive findings on the NR's ability to remodel the muscle tissue and potential NAD-dependent mechanisms. Ultimately, as we evidenced previously (12), NR supplementation showed a state-dependent effect in relation to time and diets on the caloric intake of the animals. However, although NR modulates BW, adiposity, and, according to this study, SMT, we have no clear evidence that this is the result of a change in food intake. For example, recent evidence shows that NR supplementation does not alter food intake in rodents (29–31).

At the CNS level, we found that NR was able to reverse CR-induced high TNF-α levels in the hypothalamus. Although preclinical studies have reported central antiinflammatory effects of NR (31–34), our study is the first to determine the NR effect at the hypothalamic level in an obesity and CR model. Direct suppressive effects on TNF-α levels and pathways, centrally (31–34) and peripherally (35–38), have been demonstrated in recent studies with NR. Besides, data from Roboon et al. (32) indicate that in rodents, NR inhibits neuroinflammation through suppression in CD38 ectoenzyme activity of microglial cells. Microglia are macrophage-like CNS cells that mediate inflammation in virtually all brain regions, and CD38 activity on these cells is a precipitating mechanism for neuroinflammation (39, 40). Therefore, blockade in CD38 activity in hypothalamic microglial cells may be supposedly a pathway through which, in our study, NR has reversed the neuroinflammatory status in rats under CR. Furthermore, NR-like NAD precursors have been posed as promising therapeutic strategies against neuroinflammation (41, 42).

Regardless of having normalized central TNF-α levels in CR animals, NR did not prevent muscle wasting. Anyway, this does not make it irrelevant or ineffective for SMT. It is important to emphasize that a generalized weight loss commonly affects body composition; it is estimated that in every 8–10% BW loss induced by dietary modulation, ~2–10% of muscle mass is negatively affected (43). Here, when compared to animals in the non-supplemented CR group, NR supplementation increased the BW loss by about 517%. For this reason, we expected that animals on CR supplemented with NR had a lower muscle weight compared with their control peers. Notably, this did not happen and even showed that muscle weight tended to be higher in this group of animals. Hence, we reinforce our view that NR can preserve SMT, especially during CR weight loss processes, and that its negative regulation on TNF-α may have contributed to this effect. Mechanisms other than those involving central TNF-α are also not discarded, for example, encompassing actions on muscular oxidative stress (OS).

Besides being recognized as antiinflammatory agents (41, 42), NAD precursors are also strong antioxidants (44). In our previous results (12), we showed that NR has this capacity in cardiac muscle tissue of obese rats with and without CR. On the other hand, CR plays a dichotomous role on OS; depending on the severity and/or duration, it may have either antioxidant or prooxidant activity (45). In SMT, OS triggers cellular catabolic pathways involved in muscle wasting such as autophagy, ubiquitination, and apoptosis (46). Hereby, hypothetically, it is reasonable that somehow CR would precipitate the OS process and, consequently, promote the loss of muscle mass, whereas NR, being a potential antioxidant, could modulate this scenario by dampening it. In view of this, future molecular assays are needed for a better understanding of the potential interconnections between NR, OS, CR, and SMT.

Nicotinamide riboside has been recently tested for obesity in clinical trials, showing to be safe and well-tolerated (11). However, studies have shown limited and inconclusive results for body composition and metabolism (11, 47). Further, few studies have combined NR supplementation with standard weight loss practices; only four have been found published (three in rodents and one in humans), all associated solely with physical activity (27, 48–50). Additionally, evidence for its effect on muscle tissue from obese/overweight individuals follows the same dilemma. At the same time, in the CNS, NR-led neuromodulatory and neuroprotective effects have been extremely interesting (10, 11). In this way, investigations in the line of neuropsychiatric disorders such as those that directly impact weight and body composition, especially bulimia and anorexia, are very promising.

From the current data, we want to raise the following key points for future studies involving NR, mainly on BW and body composition: (1) Benefits of BW reduction are directly linked to decreased body fat (43); (2) CR reduces BW and adiposity but usually causes fat-free lean mass loss, including SMT (2, 43); (3) preserved SMT potentiates oxidation and fat mobilization, increases basal energy expenditure, and improves physical fitness (46); (4) dietary compounds that prevent muscle catabolism in the weight loss process should be concomitantly encouraged and studied in CR therapies (8); and (5) currently, there are no human studies involving NR in combination with CR. Further reinforcing, to date, in the clinical.trials.gov platform, from 64 registered studies involving NR, none are intended for use in combination with this dietary strategy. Finally, since the results of this study are preliminary and new, naturally some insights into the NR will remain open. To soften, we discuss our findings to draw attention to the limitations and propose possible mechanisms of action, in addition to raising the importance of new preclinical and clinical investigations.

In conclusion, we show that caloric restriction in obese rats implies loss of muscle mass, observed by a reduction in the weight of the gastrocnemius muscle and neuroinflammation, evidenced by increased TNF-α levels in the hypothalamus. On the other hand, NR supplementation combined with caloric restriction suppressed neuroinflammation, enhanced BW loss, and did not alter muscle weight. Nevertheless, potential effects and mechanisms of NR for weight loss processes must be the focus of further clinical and experimental studies, thereby providing definitive information on its use as a therapeutic option for skeletal muscle health in conditions related to mass loss.

## Data Availability Statement

The raw data supporting the conclusions of this article will be made available by the authors, without undue reservation.

## Ethics Statement

The animal study was reviewed and approved by Comissão de Ética no Uso de Animais - Grupo de Pesquisa e Pós-Graduação–Hospital de Clínicas de Porto Alegre - 2018.0049.

## Author Contributions

JdC did the experimental work, statistical analysis, wrote the draft of the manuscript, and participated in the conception and design of the study. DS participated in the execution of the experiment and contributed to the writing and critical review of the final version of the manuscript for publication. HM and CdO assisted in the execution of the experiments and data collection. IT was the coordinator of the study, responsible for the conception and experimental design, critical review, technical assistance, and search for financial support. All authors contributed substantially to the execution of this study and approved the final version of this manuscript.

## Funding

This research was supported by the following Brazilian funding agencies: the Research Support Foundation of the State of Rio Grande do Sul—FAPERGS (JdC); the Coordination for the Improvement of Higher Education Personnel—CAPES (DS); the National Council for Scientific and Technological Development—CNPq (IT); and the Graduate Research Group of Hospital de Clínicas de Porto Alegre (GPPG/HCPA) through the Research Incentive Fund (FIPE) (IT—Grant #18-0049).

## Conflict of Interest

The authors declare that the research was conducted in the absence of any commercial or financial relationships that could be construed as a potential conflict of interest.

## Publisher's Note

All claims expressed in this article are solely those of the authors and do not necessarily represent those of their affiliated organizations, or those of the publisher, the editors and the reviewers. Any product that may be evaluated in this article, or claim that may be made by its manufacturer, is not guaranteed or endorsed by the publisher.

## References

[1] Blüher M. Obesity: global epidemiology and pathogenesis. Nat Rev Endocrinol. (2019) 15:288–98. 10.1038/s41574-019-0176-8 30814686

[2] Redman LM Ravussin E. Caloric restriction in humans: impact on physiological, psychological, and behavioral outcomes. Antioxid Redox Signal. (2011) 14:275–87. 10.1089/ars.2010.3253 20518700 PMC3014770

[3] Vaughan KL Kaiser T Peaden R Anson RM de Cabo R Mattison JA. Caloric restriction study design limitations in rodent and nonhuman primate studies. J Gerontol A Biol Sci Med Sci. (2017) 73:48–53. 10.1093/gerona/glx088 28977341 PMC5861872

[4] Mukund K Subramaniam S. Skeletal muscle: a review of molecular structure and function, in health and disease. Wiley Interdiscip Rev Syst Biol Med. (2020) 12:e1462. 10.1002/wsbm.1462 31407867 PMC6916202

[5] Delezie J Handschin C. Endocrine crosstalk between skeletal muscle and the brain. Front Neurol. (2018) 9:698. 10.3389/fneur.2018.00698 30197620 PMC6117390

[6] Duan K Gao X Zhu D. The clinical relevance and mechanism of skeletal muscle wasting. Clin Nutr. (2021) 40:27–37. 10.1016/j.clnu.2020.07.029 32788088

[7] Burfeind KG Michaelis KA Marks DL. The central role of hypothalamic inflammation in the acute illness response and cachexia. Semin Cell Dev Biol. (2016) 54:42–52. 10.1016/j.semcdb.2015.10.038 26541482 PMC4872506

[8] Willoughby D Hewlings S Kalman D. Body composition changes in weight loss: strategies and supplementation for maintaining lean body mass, a brief review. Nutrients. (2018) 10:1876. 10.3390/nu10121876 30513859 PMC6315740

[9] Yang Y Sauve AA. NAD(+) metabolism: bioenergetics, signaling and manipulation for therapy. Biochim Biophys Acta. (2016) 1864:1787–800. 10.1016/j.bbapap.2016.06.014 27374990 PMC5521000

[10] Chi Y Sauve AA. Nicotinamide riboside, a trace nutrient in foods, is a Vitamin B3 with effects on energy metabolism and neuroprotection. Curr Opin Clin Nutr Metab Care. (2013) 16:657–61. 10.1097/MCO.0b013e32836510c0 24071780

[11] Mehmel M Jovanović N Spitz U. Nicotinamide riboside-the current state of research and therapeutic uses. Nutrients. (2020) 12:1616. 10.3390/nu12061616 32486488 PMC7352172

[12] de Castro JM Assumpção JAF Stein DJ Toledo RS da Silva LS Caumo W. Nicotinamide riboside reduces cardiometabolic risk factors and modulates cardiac oxidative stress in obese Wistar rats under caloric restriction. Life Sci. (2020) 263:118596. 10.1016/j.lfs.2020.118596 33080243

[13] Percie du Sert N Ahluwalia A Alam S Avey MT Baker M Browne WJ. Reporting animal research: explanation and elaboration for the ARRIVE guidelines 2.0. PLoS Biol. (2020) 18:e3000411. 10.1371/journal.pbio.3000411 32663221 PMC7360025

[14] Sampey BP Vanhoose AM Winfield HM Freemerman AJ Muehlbauer MJ Fueger PT. Cafeteria diet is a robust model of human metabolic syndrome with liver and adipose inflammation: comparison to high-fat diet. Obesity. (2011) 19:1109–17. 10.1038/oby.2011.18 21331068 PMC3130193

[15] Gariani K Menzies KJ Ryu D Wegner CJ Wang X Ropelle ER. Eliciting the mitochondrial unfolded protein response by nicotinamide adenine dinucleotide repletion reverses fatty liver disease in mice. Hepatology. (2016) 63:1190–204. 10.1002/hep.28245 26404765 PMC4805450

[16] Cantó C Houtkooper RH Pirinen E Youn DY Oosterveer MH Cen Y. The NAD(+) precursor nicotinamide riboside enhances oxidative metabolism and protects against high-fat diet-induced obesity. Cell Metab. (2012) 15:838–47. 10.1016/j.cmet.2012.04.022 22682224 PMC3616313

[17] Bradford MM. A rapid and sensitive method for the quantitation of microgram quantities of protein utilizing the principle of protein-dye binding. Anal Biochem. (1976) 72:248–54. 10.1016/0003-2697(76)90527-3 942051

[18] Moroz N Carmona JJ Anderson E Hart AC Sinclair DA Blackwell TK. Dietary restriction involves NAD^+^ -dependent mechanisms and a shift toward oxidative metabolism. Aging Cell. (2014) 13:1075–85. 10.1111/acel.12273 25257342 PMC4244309

[19] Kitada M Ogura Y Monno I Koya D. The impact of dietary protein intake on longevity and metabolic health. EBioMedicine. (2019) 43:632–40. 10.1016/j.ebiom.2019.04.005 30975545 PMC6562018

[20] Becher B Spath S Goverman J. Cytokine networks in neuroinflammation. Nat Rev Immunol. (2017) 17:49–59. 10.1038/nri.2016.123 27916979

[21] Iyer SS Cheng G. Role of interleukin 10 transcriptional regulation in inflammation and autoimmune disease. Crit Rev Immunol. (2012) 32:23–63. 10.1615/critrevimmunol.v32.i1.30 22428854 PMC3410706

[22] Cuspidi C Rescaldani M Sala C Grassi G. Left-ventricular hypertrophy and obesity: a systematic review and meta-analysis of echocardiographic studies. J Hypertens. (2014) 32:16–25. 10.1097/HJH.0b013e328364fb58 24309485

[23] Seldeen KL Shahini A Thiyagarajan R Redae Y Leiker M Rajabian N. Short-term nicotinamide riboside treatment improves muscle quality and function in mice and increases cellular energetics and differentiating capacity of myogenic progenitors. Nutrition. (2021) 87–88:111189. 10.org/10.1016/j.nut.2021.111189 33744645 PMC8713751

[24] Poljsak B Milisav I. Vitamin B3 forms as precursors to NAD+: are they safe? Trends Food Sci Technol. (2018) 79:198–203. 10.1016/j.tifs.2018.07.020

[25] Goody MF Henry CA. A need for NAD+ in muscle development, homeostasis, and aging. Skelet Muscle. (2018) 8:9. 10.1186/s13395-018-0154-1 29514713 PMC5840929

[26] Remie CME Roumans KHM Moonen MPB Connell NJ Havekes B Mevenkamp J. Nicotinamide riboside supplementation alters body composition and skeletal muscle acetylcarnitine concentrations in healthy obese humans. Am J Clin Nutr. (2020) 112:413–26. 10.1093/ajcn/nqaa072 32320006 PMC7398770

[27] Crisol BM Veiga CB Braga RR Lenhare L Baptista IL Gaspar RC. NAD+ precursor increases aerobic performance in mice. Eur J Nutr. (2020) 59:2427–37. 10.1007/s00394-019-02089-z 31494696

[28] Frederick DW Loro E Liu L Davila A Chellappa K Silverman IM. Loss of NAD homeostasis leads to progressive and reversible degeneration of skeletal muscle. Cell Metab. (2016) 24:269–82. 10.1016/j.cmet.2016.07.005 27508874 PMC4985182

[29] Shi W Hegeman MA Doncheva A Bekkenkamp-Grovenstein M de Boer VCJ Keijer J. High dose of dietary nicotinamide riboside induces glucose intolerance and white adipose tissue dysfunction in mice fed a mildly obesogenic diet. Nutrients. (2019) 11:439. 10.3390/nu11102439 31614949 PMC6835358

[30] Shi W Hegeman MA Doncheva A van der Stelt I Bekkenkamp-Grovenstein M van Schothorst EM. Transcriptional response of white adipose tissue to withdrawal of vitamin B3. Mol Nutr Food Res. (2019) 63:e1801100. 10.1002/mnfr.201801100 30990964 PMC6618275

[31] Jiang Y Liu Y Gao M Xue M Wang Z Liang H. Nicotinamide riboside alleviates alcohol-induced depression-like behaviours in C57BL/6J mice by altering the intestinal microbiota associated with microglial activation and BDNF expression. Food Funct. (2020) 11:378–91. 10.1039/c9fo01780a 31820774

[32] Roboon J Hattori T Ishii H Takarada-Iemata M Nguyen DT Heer CD. Inhibition of CD38 and supplementation of nicotinamide riboside ameliorate lipopolysaccharide-induced microglial and astrocytic neuroinflammation by increasing NAD+. J Neurochem. (2021) 158:311–27. 10.1111/jnc.15367 33871064 PMC8282715

[33] Lee HJ Yang SJ. Supplementation with nicotinamide riboside reduces brain inflammation and improves cognitive function in diabetic mice. Int J Mol Sci. (2019) 20:196. 10.3390/ijms20174196 31461911 PMC6747453

[34] Joshi U Evans JE Pearson A Saltiel N Cseresznye A Darcey T. Targeting sirtuin activity with nicotinamide riboside reduces neuroinflammation in a GWI mouse model. Neurotoxicology. (2020) 79:84–94. 10.1016/j.neuro.2020.04.006 32343995

[35] Lee HJ Hong Y-S Jun W Yang SJ. Nicotinamide riboside ameliorates hepatic metaflammation by modulating NLRP3 inflammasome in a rodent model of type 2 diabetes. J Med Food. (2015) 18:1207–13. 10.1089/jmf.2015.3439 25974041

[36] Elhassan YS Kluckova K Fletcher RS Schmidt MS Garten A Doig CL. Nicotinamide riboside augments the aged human skeletal muscle NAD(+) metabolome and induces transcriptomic and anti-inflammatory signatures. Cell Rep. (2019) 28:1717–28.e6. 10.1016/j.celrep.2019.07.043 31412242 PMC6702140

[37] de Oliveira C de Freitas JS Macedo IC Scarabelot VL Ströher R. Transcranial direct current stimulation (tDCS) modulates biometric and inflammatory parameters and anxiety-like behavior in obese rats. Neuropeptides. (2019) 73:1–10. 10.1016/j.npep.2018.09.006 30446297

[38] Lee HJ Yang SJ. Nicotinamide riboside regulates inflammation and mitochondrial markers in AML12 hepatocytes. Nutr Res Pract. (2019) 13:319cy 10.4162/nrp.2019.13.1.3 30788050 PMC6369115

[39] Roboon J Hattori T Ishii H Takarada-Iemata M Le TM Shiraishi Y. Deletion of CD38 suppresses glial activation and neuroinflammation in a mouse model of demyelination. Front Cell Neurosci. (2019) 13:258. 10.3389/fncel.2019.00258 31244614 PMC6563778

[40] Guerreiro S Privat A-L Bressac L Toulorge D. CD38 in neurodegeneration and neuroinflammation. Cells. (2020) 9:471. 10.3390/cells9020471 32085567 PMC7072759

[41] Braidy N Berg J Clement J Khorshidi F Poljak A Jayasena T. Role of nicotinamide adenine dinucleotide and related precursors as therapeutic targets for age-related degenerative diseases: rationale, biochemistry, pharmacokinetics, and outcomes. Antioxid Redox Signal. (2019) 30:251–94. 10.1089/ars.2017.7269 29634344 PMC6277084

[42] Lautrup S Sinclair DA Mattson MP Fang EF. NAD(+) in brain aging and neurodegenerative disorders. Cell Metab. (2019) 30:630–55. 10.1016/j.cmet.2019.09.001 31577933 PMC6787556

[43] Cava E Yeat NC Mittendorfer B. Preserving healthy muscle during weight loss. Adv Nutr. (2017) 8:511–9. 10.3945/an.116.014506 28507015 PMC5421125

[44] Massudi H Grant R Guillemin GJ Braidy N. NAD+ metabolism and oxidative stress: the golden nucleotide on a crown of thorns. Redox Rep. (2012) 17:28–46. 10.1179/1351000212Y.0000000001 22340513 PMC6837626

[45] Walsh ME Shi Y Van Remmen H. The effects of dietary restriction on oxidative stress in rodents. Free Radic Biol Med. (2014) 66:88–99. 10.1016/j.freeradbiomed.2013.05.037 23743291 PMC4017324

[46] Powers SK Kavazis AN DeRuisseau KC. Mechanisms of disuse muscle atrophy: role of oxidative stress. Am J Physiol Integr Comp Physiol. (2005) 288:R337–44. 10.1152/ajpregu.00469.2004 15637170

[47] Fluharty NT Brenner C. Fat mobilization without weight loss is a potentially rapid response to nicotinamide riboside in obese people: it's time to test with exercise. Am J Clin Nutr. (2020) 112:243–4. 10.1093/ajcn/nqaa109 32412605 PMC7398765

[48] Kourtzidis IA Dolopikou CF Tsiftsis AN Margaritelis N V Theodorou AA Zervos IA. Nicotinamide riboside supplementation dysregulates redox and energy metabolism in rats: implications for exercise performance. Exp Physiol. (2018) 103:1357–66. 10.1113/EP086964 30007015

[49] Stocks B Ashcroft SP Joanisse S Dansereau LC Koay YC Elhassan YS. Nicotinamide riboside supplementation does not alter whole-body or skeletal muscle metabolic responses to a single bout of endurance exercise. J Physiol. (2021) 599:1513–31. 10.1113/JP280825 33492681

[50] Kourtzidis IA Stoupas AT Gioris IS Veskoukis AS Margaritelis N V Tsantarliotou M. The NAD+ precursor nicotinamide riboside decreases exercise performance in rats. J Int Soc Sports Nutr. (2016) 13:32. 10.1186/s12970-016-0143-x 27489522 PMC4971637

