# Supplementary figures and images for: Nicotinamide Riboside Neutralizes Hypothalamic Inflammation and Increases Weight Loss Without Altering Muscle Mass in Obese Rats Under Calorie Restriction: A Preliminary Investigation
Source: Front Nutr. 2021 Sep 13;8:648893. doi: 10.3389/fnut.2021.648893 (PMC8475757; doi:10.3389/fnut.2021.648893)

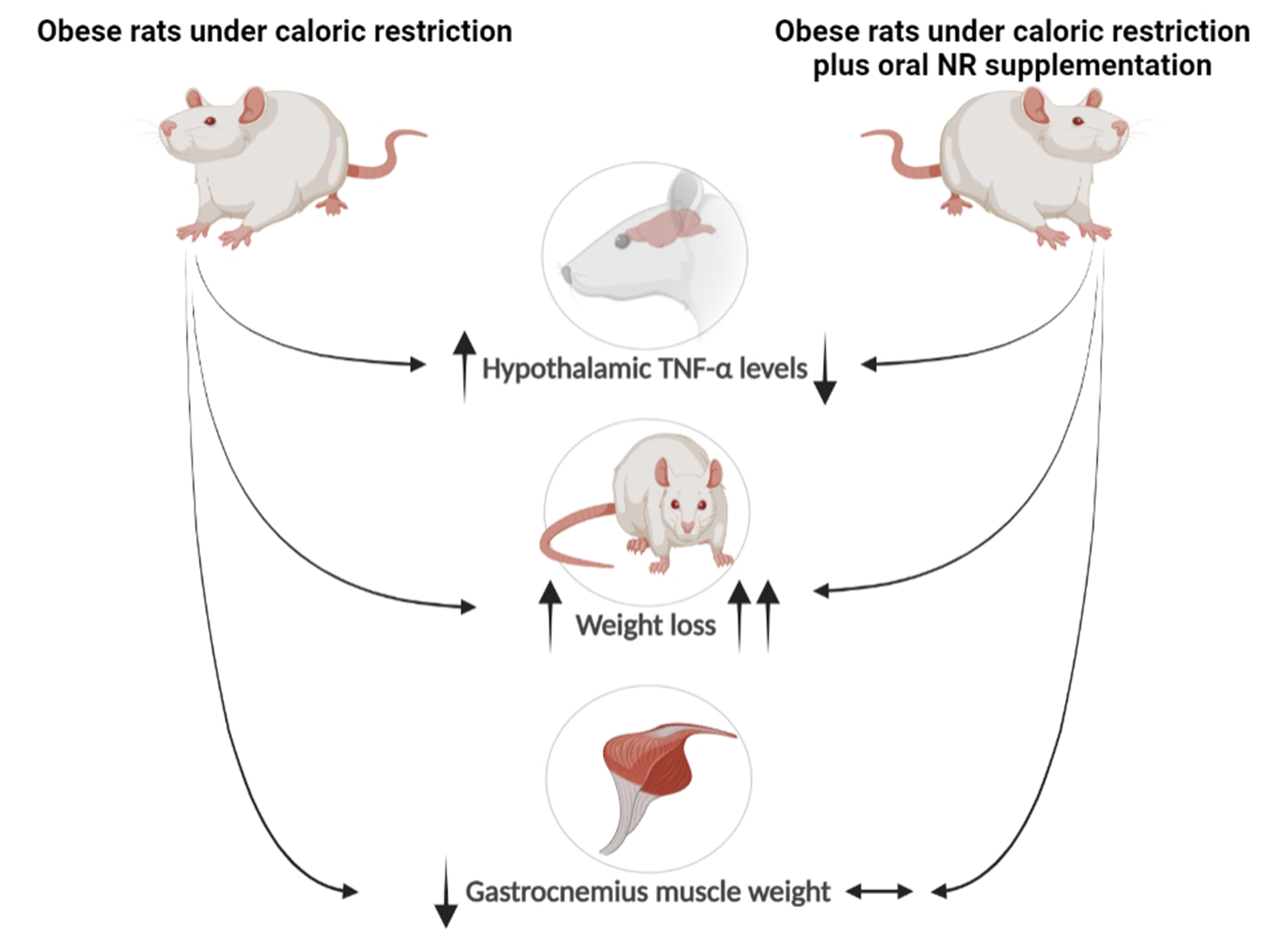

Supplement: Supplementary file 1 [file Image_1.JPEG]
