# Supplementary material for: Nicotinamide Riboside Neutralizes Hypothalamic Inflammation and Increases Weight Loss Without Altering Muscle Mass in Obese Rats Under Calorie Restriction: A Preliminary Investigation
Source: Front Nutr. 2021 Sep 13;8:648893. doi: 10.3389/fnut.2021.648893 (PMC8475757; doi:10.3389/fnut.2021.648893)
Supplement: Supplementary file 2 [file Table_1.DOCX]

***Supplementary Material***

# Supplementary Figure


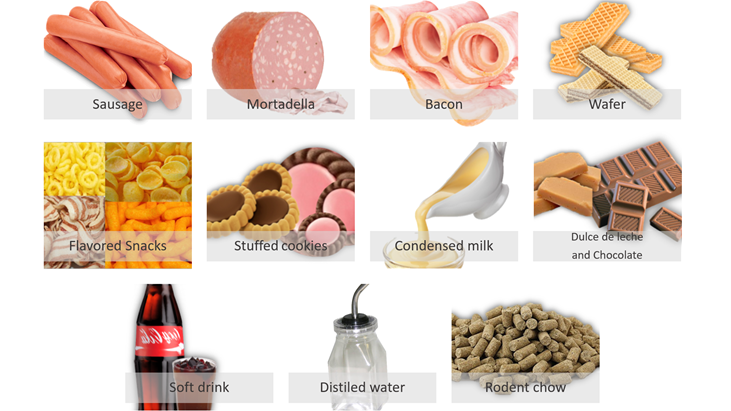


**Supplementary Figure 1.** Images illustrating foods offered in the cafeteria diet.

# Supplementary Table

| **Food type** | **Food brand** | **Energy**  **(kJ=kcal)** | **Total Fat**  **(g)** | **Saturated Fat**  **(g)** | **Trans Fat**  **(g)** | **Cholesterol**  **(mg)** | **Sodium**  **(mg)** | **Carbohydrate**  **(g)** | **Dietary Fiber**  **(g)** | **Protein**  **(g)** |
| --- | --- | --- | --- | --- | --- | --- | --- | --- | --- | --- |
| Stuffed cookies | Tortinhas Chocolate - Isabela® | 209.2=50.00 | 2.33 | 1.20 | 0.00 | * | 27.60 | 6.67 | 0.20 | 0.73 |
|  | Tortinhas *Due Trufa* e Geleia de Morango - Isabela® | 207.8=49.67 | 2.27 | 0.53 | 0.00 | * | 19.60 | 6.67 | 0.20 | 0.67 |
| Wafer | Wafer morango - Isabela® | 220.4=52.67 | 2.83 | 1.50 | 0.00 | * | 14.40 | 6.33 | 0.20 | 0.50 |
|  | Wafer chocolate - Isabela® | 212.0=50.67 | 2.17 | 1.27 | 0.00 | * | 17.60 | 6.33 | 0.20 | 0.60 |
| Dulce de leche | Doce de Leite - DaColonia® | 151.0=36.10 | 0.45 | 0.25 | 0.00 | * | 8.00 | 7.50 | 0.00 | 1.01 |
| Chocolate | Chocolate ao leite - Lacta® | 213.8=51.10 | 2.65 | 1.60 | 0.00 | * | 11.0 | 6.50 | 0.00 | 0.35 |
| Condensed milk | Leite condensado - Italac® | 136.0=32.50 | 0.80 | 0.50 | 0.00 | * | 18.00 | 5.50 | 0.00 | 0.75 |
| Soft drink | Refrigerante de cola - Coca-Cola® | 17.8=4.26 | 0.00 | 0.00 | 0.00 | * | 0.50 | 1.05 | 0.00 | 0.00 |
| Flavored snacks | Salgadinho de milho - Cheetos® Lua | 199.6=47.70 | 2.24 | 0.52 | 0.00 | 0.00 | 48.00 | 6.40 | 0.00 | 0.60 |
|  | Salgadinho de milho - Fandangos® Queijo | 205.9=49.20 | 2.28 | 0.40 | 0.00 | * | 58.40 | 6.80 | 0.00 | 0.56 |
|  | Salgadinho de trigo - Baconzitos ® | 229.7=54.90 | 3.70 | 2.11 | 0.00 | * | 52.00 | 4.80 | 0.00 | 0.60 |
|  | Salgadinho de milho - Cebolitos® | 215.9=51.60 | 2.68 | 1.12 | 0.00 | * | 74.00 | 6.40 | 0.00 | 0.48 |
| Hot dog sausage | Salsicha tradicional - Excelsior® | 90.4=21.60 | 1.62 | 0.46 | 0.00 | * | 111.80 | 0.44 | 0.00 | 1.32 |
| Mortadella | Mortadela - Aurora® | 106.7=25.50 | 2.00 | 0.85 | 0.00 | * | 141.50 | 0.60 | 0.00 | 1.30 |
| Bacon | Bacon - Frimesa® | 125.5=30.00 | 2.40 | 0.90 | 0.00 | * | 90.00 | 0.00 | 0.00 | 2.00 |

**Supplementary Table 1.** Individual nutritional data of foods included in the cafeteria diet. **Table legend:** Values equivalent to a 10 gram portion. Nutritional values calculated based on commercial product information. Acronyms and symbols: kcal=kilocalorie; kJ=kilojoule; g=gram; mg=milligram; *=unreported or established values; ®=registered trademark. Acronyms and symbols: kcal=kilocalorie; kJ=kilojoule; g=gram; mg=milligram; *=unreported or established values.

| **Vitamins** | |
| --- | --- |
| Alpha-tocopherol (Vit E) | 34 Ul/kg |
| Biotin (Vit B7) | 0.05 mg/kg |
| Cholecalciferol (Vit D3) | 2.000 Ul/kg |
| Choline chloride | 1.900 mg/kg |
| Cobalamin (Vit B12) | 22 mcg/kg |
| Folic acid (Vit B9) | 1 mg/kg |
| Menadione (Vit K3) | 3 mg/kg |
| Niacin (Vit B3) | 60 mg/kg |
| Pyridoxine (Vit B6) | 7 mg/kg |
| Retinol (Vit A) | 13.000 Ul/kg |
| Riboflavin (Vit B2) | 6 mg/kg |
| Thiamine (Vit B1) | 5 mg/kg |
| **Minerals** | |
| Calcium | 10-14 g/kg |
| Cobalt | 1.5 mg/kg |
| Copper | 10 mg/kg |
| Fluoride | 80 mg/kg |
| Iodine | 2 mg/kg |
| Iron | 50 mg/kg |
| Manganese | 60 mg/kg |
| Phosphorus | 8.000 mg/kg |
| Selenium | 0.05 mg/kg |
| Sodium | 2.700 mg/kg |
| Zinc | 60 mg/kg |
| **Specific amino acids** | |
| Lysine | 12 g/kg |
| Methionine | 4.000 mg/kg |
| **Additives** | |
| BHT | 100 mg/kg |
| **Constituent ingredients** | |
| Ground whole corn, Soybean bran, Wheat bran, Calcium carbonate, Dicalcium phosphate, Sodium chloride, Retinol (Vit A), Cholecalciferol (Vit D3), Alpha-tocopherol (Vit E), Menadione (Vit K3), Thiamine (Vit B1), Riboflavin (Vit B2), Pyridoxine (Vit B6), Cobalamin (Vit B12), Niacin (Vit B3), Calcium pantothenate, Folic acid (Vit B9), Biotin (Vit B7), Vegetable oil, Choline chloride, Manganese sulfate, Iron sulfate, Zinc sulfate, Copper sulfate, Calcium iodate, Manganese sulfate, Cobalt sulfate, Lysine,Methionine, Butylated hydroxytoluene (BHT). | |

**Supplementary Table 2.** General nutritional information on rodent chow. Table legend: Values equivalent to a 1000 gram portion (or 1 kilogram). Nutritional values calculated based on commercial product information. Acronyms and symbols: Ul= international units; mg=milligrams; g=grams; mcg=microgram
